# Communication skills utilized by physicians in the pediatric outpatient setting

**DOI:** 10.1186/s12913-022-08385-5

**Published:** 2022-08-04

**Authors:** T. Lee, E. C. Lin, H. C. Lin

**Affiliations:** 1 Division of Gastroenterology, Hepatology, and Nutrition, Children’s Hospital of Philadelphia, Philadelphia, PA USA; 2 Philadelphia College of Osteopathic Medicine, Philadelphia, PA USA; 3 Department of Medicine, Oregon Health & Sciences University, Portland, OR USA; 4 Department of Pediatrics, Oregon Health & Sciences University, Portland, OR USA

**Keywords:** Pediatrics, Communication, Patient satisfaction, Communication strategies

## Abstract

**Background:**

Effective communication has been shown to increase patient satisfaction. The objective of this study was to describe communication strategies employed by physicians, and determine if physician communication strategies affect caregiver perception of quality or satisfaction with physician communication in a pediatric ambulatory setting.

**Methods:**

This observational study was conducted at the Children’s Hospital of Philadelphia and consisted of video recordings of visits that were reviewed by research assistants for physician utilized communication strategies. Caregivers completed surveys on their preferred physician communication qualities, perception of communication quality, and satisfaction with communication. Correlation was performed between types of communication strategy and caregiver satisfaction with communication or perceived quality of communication. T-tests were run to see if there was a significant difference in patient perceived communication and satisfaction scores based on the communication strategies utilized during visits.

**Results:**

There were five universally used communication strategies across the 84 clinic visits recorded, including: eye contact, good posture, speaking concisely, providing thorough explanations, and providing summary of next steps. The average number of communication strategies used was 15.95 (σ = 1.50) with physicians using at least 16 of the 18 communication strategies in 62% of the clinic visits. There was no correlation between the number of communication strategies physicians utilized and either the caregiver perception of communication quality score (CPCQ) or communication satisfaction (CS) score. Caregivers who preferred an authoritative approach but perceived a collaborative approach reported lower average CPCQ and CS scores compared to caregivers who had their communication expectations met.

**Discussion:**

There are numerous tools designed to help the physician facilitate an effective working relationship with the patient. In our study, the universally used verbal communication strategies are generally recognized as components of an effective communication repertoire. Another part of effective communication is meeting communication expectations with the CS scores suggesting that caregivers felt their communication needs were being met. Dedicating clinical time to understanding this need may help improve the overall clinical experience.

**Conclusion:**

Physicians utilize many of the suggested communication strategies to help facilitate an effective clinical encounter. Further studies on caregiver communication requirements and meeting caregiver communication expectations are needed.

## Background

Effective communication is key to providing high quality care as it allows the healthcare provider to solicit the relevant medical history to understand the patient’s concerns, generate a differential, and then facilitate a discussion to engage the family in a shared decision-making process on next steps in management. As such, interpersonal and communication skills are one of the Accreditation Council for Graduate Medical Education (ACGME) core competencies with the goal for all physicians to communicate effectively both with patients and families, as well as with health professionals [1]. These communication skills include a combination of both verbal and non-verbal skills to help the physician establish and sustain an effective working relationship with the patient. Central to this relationship is the development of trust with the patient and as such, there is an emphasis on interpersonal communication in medical education [1–3].

As physicians in a teaching hospital, it is important to model appropriate communication behavior for trainees.

Numerous studies have demonstrated the benefits of the patient communication aspect of this core competency. Patient satisfaction is often used as a measure of provider communication skills and generally, literature has shown that patient centered communication leads to higher rates of patient satisfaction [4–9]. Non-verbal communication skills play an equal, if not more significant role than technical verbal communication skills. Facial expressions, affirmative gestures, unpurposive movements, and hand gestures had a significant positive influence on patient satisfaction and perception of interviews with their physicians [10, 11].

Teaching and development of these communication skills typically start during medical school and can involve a myriad of approaches. In a study using the Calgary-Cambridge guide to teach students basic communication and counseling skills, about 90% of students showed marked improvement in communication, as shown by their improved standardized patient satisfaction questionnaire after training [12, 13]. Video recording students and providing feedback using mock patient interactions is another method medical schools use to teach effective communication skills with one study reporting marked improvement in “identification convergence, information seeking, information giving, and nonverbal behaviors” [14], and another demonstrating that medical students improving their overall interview scores after receiving feedback from their preceptors [15]. Modeling is another form of teaching that has been shown to benefit future physician’s communication skills. Shadowing and observing physician role models have been shown to significantly influence learners as they copy their instructor’s behavior (consciously and unconsciously) [16, 17]. Other methods like role play and didactic learning have been utilized in the teaching of effective communication with patients [17]. Ultimately, teaching effective communication skills to medical students has been proven to improve physician patient interactions and patient outcomes with both verbal and non-verbal communication essential to effective patient encounters [18]. Currently, medical training remains focused on training learners in traditional communication strategies by utilizing training rubrics such as the Kalamazoo Essential Elements Communication Checklist (KEECC) [17] or the Calgary-Cambridge Guide.

The objectives of this study are to: 1) describe communication strategies employed by physicians in an outpatient setting, and 2) determine if select communication strategies affect caregiver perception or satisfaction with physician communication.

## Methods

An observational study was performed at the Children’s Hospital of Philadelphia outpatient gastroenterology clinic from March 1, 2016 to October 31, 2016. Ethics approval was reviewed and approved by the Children’s Hospital of Philadelphia’s Institutional Review Board. The study consisted of video recording outpatient clinical encounters with caregiver participants also completing a post-visit survey on their perception of quality of communication and satisfaction with communication. This was a sample of convenience for participation in the study and consent was obtained from caregivers, patients, and the physician. All participants had the option of stopping the video recording any time during the visit.

Research assistants were trained in identifying physician communication behaviors based on the Kalamazoo Essential Elements Communications Checklist (KEECC), which is a validated measure of communication strategies used in the clinical encounter [19, 20]. The KEECC is consists of a framework of seven communication tasks that has been used as a communication standard in medicine. These communication tasks include: build the relationship, open the discussion, gather information, understand the patient’s perspective, share information; reach agreement, and provide closure. Each video recording of the visit was reviewed by 3 different research assistants for physician employed communication behaviors based on the KEECC. In addition, the research assistants reviewed the video of each clinic visit to assess the following: 1) if the patient or caregiver was the primary speaker, and 2) if the physician primarily interacted with the patient or caregiver. Differences in observations between research assistants were discussed and reviewed.

The post-visit survey responses were based on a 5-point Likert scale to measure caregiver perception of communication quality (CPCQ) based on the KEECC and communication satisfaction (CS) based on a validated modified version focusing on communication specific statements of the Patient Satisfaction Questionnaire 18 [21, 22]. Survey responses were analyzed and descriptive statistics were used to summarize communication strategies used. Correlation was performed between types of communication strategy and caregiver satisfaction with communication or perceived quality of communication. For quantitative analysis, t-tests were used to assess differences in patient perceived communication and satisfaction scores based on the communication strategies utilized during visits.

## Results

A total of 84 participants were consented for study with their clinical visits were video recorded. The clinical visits were conducted by 7 different providers. The caregiver of the patient was the primary speaker during 90.5% (*n* = 76) of the clinical visits, with the child being the primary speaker in the other 8 visits. In the visits in which the caregiver was the primary speaker, the physician primarily interacted with the caregiver in 82.9% (*n* = 63) visits, and equally with both the child and caregiver in 17.1% of the visits. For the visits with the patient being the primary communicator, the physician primarily interacted with the patient in only 1 of the visits (12.5%), primarily with both the patient and caregiver in 62.5% and primarily with the caregiver in 25% of the visits.

The average caregiver perceived communication quality (CPCQ) score was 4.61 out of 5 (σ = 0.63). For visits in which the caregiver was the primary communicator, caregivers rated the quality of communication to be 4.60 (σ = 0.62), compared to an average of 4.71 (σ = 0.76) in visits where the patient was the primary communicator. The average communication satisfaction (CS) score was 4.69 out of 5 (σ = 0.60). For visits in which the caregiver was the primary communicator, the communication satisfaction was rated to 4.69 (σ = 0.59), compared to an average of 4.71 (σ = 0.76) in visits where the patient was the primary communicator (Table 1).

**Table 1:** Communication quality and satisfaction by primary communicator

Primary Communicator	Patient Age Range (years)	Patient Median Age (years)	CPCQ^a^ Score	CS^b^ Score
Parent (Mother or Father)	0–17	6	4.60 ± 0.62	4.69 ± 0.59
Patient	10–16	13	4.71 ± 0.76	4.71 ± 0.76
Overall	0–17	7	4.61 ± 0.63	4.69 ± 0.60

^a^ *CPCQ* Caregiver perceived communication quality (CPCQ) is based off of the Kalamazoo Essential Elements of Communication Checklist

^b^ *CS* Communication satisfaction (CS) score is based off of a modified version of the Patient Satisfaction Questionnaire-18

**Fig. 1:**
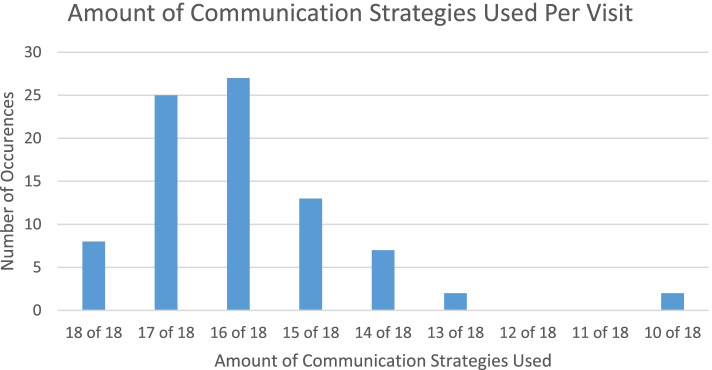
Amount of communication strategies used per visit

On review of the recorded visits for communication strategies used during the clinical visit, the average number of KEECC communication strategies used was 15.95 (σ = 1.50). All 18 communication strategies were utilized by physicians in 9.52% (*n* = 8) of the visits, with physicians using at least 16 of the 18 communication strategies in 62% of the clinic visits (Fig. 1). There was no correlation between the number of communication strategies physicians utilized and either the caregiver perceived communication quality (CPCQ) score or the communication satisfaction (CS) score.

The top communication strategies employed by physicians during the clinical visit were eye contact, good posture, speaking concisely, providing thorough explanations, and providing summary of next steps (all used in 100% of clinical visits). The least used communication strategies were shaking hands, introducing self, explaining actions of exams, encouraging questions, and asking about others’ point of view (Table 2). There was no statistically significant difference in the caregiver perceived communication quality (CPCQ) score or the communication satisfaction (CS) score based on whether physicians utilized or did not utilize these 5 communication strategies of shaking hands, introducing self, explaining actions of exams, encouraging questions, and asking about others’ point of view.

**Table 2:** List of communication strategies used by physicians during clinical visits (*n* = 84)

Communication Strategy	Clinic Visits	Percentage Used
Eye Contact	84	100.00%
Good posture	84	100.00%
Spoke concisely	84	100.00%
Provided thorough explanations	84	100.00%
Provided summary of next steps	84	100.00%
Positive Facial Expressions	83	98.81%
Asked for clarification	83	98.81%
Washed Hands	82	97.62%
Used Neutral Tone of Voice	82	97.62%
Summarized the information	82	97.62%
Provided summary of findings	82	97.62%
Asked open-ended questions	78	92.86%
Used Hand Gestures	74	88.10%
Asked about others point of view	70	83.33%
Encouraged Questions	68	80.95%
Explained actions of exam	64	76.19%
Introduce Self	51	60.71%
Shook Hands	21	25.00%

Formal greeting and physician introductions occurred in 89.1% (*n* = 33) of new patient visits compared to only 38.3% of follow-up visits (*n* = 18) (*p*-value < 0.01). Unlike physician introductions, hand shaking was not statistically significantly different by visit type. Based on the KEECC, shaking hands and introducing self are part of establishing the relationship and on the CPCQ score, the average building a relationship category score was 4.58 out of 5 (σ = 0.71). Physicians greeted or introduced themselves to the patient or caregiver in 60.7% of the clinical visits. The physician shook hands with the patient or caregiver in only 25% of the visits. There was no statistically significant difference in the CPCQ building a relationship sub-score between visits in which the physician introduced self or shook hands, and those in which the physician did not introduce self or shake hands. There was also no statistically significant difference in the CPCQ building a relationship sub-score between visits in which the physician introduced self and shook hands versus visits in which the physician did neither.

Caregivers were surveyed on their physician communication expectations based on a statement of either: “I expect the healthcare provider to tell me/my child what to do” which suggests an authoritative approach to the physician relationship or “I like to ask questions before accepting provider recommendations” which would suggest a collaborative approach, or “other”. 40.5% (*n* = 34) reported an authoritative communication preference, while 57.1% (*n* = 48) preferred a collaborative approach. There was no statistically significant difference in either the caregiver perceived communication quality score (*p*-value 0.823) or communication satisfaction score (*p*-value 0.573) between caregivers with an authoritative versus a collaborative preference.

Of the caregivers preferring an authoritative communication preference, 70.6% reported a match in communication expectations in that they experienced a more authoritative clinical visit in which they were told about the clinical management and asked few questions. For caregivers preferring a more collaborative clinical experience, only 47.9% reported a match in communication expectations in that they asked questions and discussed about treatment options (Table 3). There was no statistically significant difference in the caregiver perceived communication quality (CPCQ) score or the communication satisfaction (CS) score between caregivers who had matched expectations compared to those with mismatched expectations (i.e., preferred a collaborative communication approach, but physician adopted a more authoritative approach or vice versa). However, for caregivers preferring an authoritative approach but perceived that they were engaged in a more collaborative discussion, they reported a lower average CS score (4.19 versus 4.81, *p*-value 0.018) and a lower average CPCQ score (4.18 versus 4.73, *p*-value 0.046) compared to caregivers who perceived a more authoritative discussion and had their communication expectations met.

**Table 3:** Communication quality and satisfaction by primary communicator

Patient Preferred Communication Style	Communication Style Met (n visits)	%	Communication Style Not Met (n visits)	%	No Answer
^a^Authoritative (*n* = 34)	24	70.6%	8	23.5%	2
^b^Collaborative (*n* = 48)	23	47.9%	18	37.5%	7

^a^Authoritative preferred communication style based off selected statement “I expect the healthcare provider to tell me/my child what to do”

^b^Collaborative preferred communication style based off selected statement “I like to ask questions before accepting provider recommendations”

The CPCQ scores on communication quality were compared with CS scores on satisfaction. There was a high correlation between the KEECC and satisfaction scores (*R* ^2^ = 0.805). The KEECC communication element that correlated the highest with the average communication satisfaction (CS) score was understanding patient perspective (*R* ^2^ = 0.758).

## Discussion

In our study, based on the KEECC, physicians were utilizing a majority of the communication strategies during the outpatient clinic visit. There were five communication strategies, including both verbal and non-verbal strategies, that we utilized in each of the visits: eye contact, good posture, speaking concisely, providing thorough explanations, and providing summary of next steps. Good eye contact and posture are both key components in non-verbal communication models in medicine such as in the KEECC or SOLER (sit squarely, open posture, lean towards the other, eye contact, relax) model for nursing communication [23]. Nonverbal communication strategies help facilitate active or participatory listening to engage the patient in shared decision making, and can also assist the physician in conveying empathy and compassion. In addition, studies report that good eye contact is associated higher patient satisfaction [24–28].

The universally used verbal communication strategies of speaking concisely, providing thorough explanations, and providing summary of next steps make are generally recognized as components of an effective communication repertoire. Speaking concisely is one strategy to minimize medical jargon and to communicate at a more easily comprehensible level for the patient. Using medical jargon or circuitous explanations presents an unnecessary barrier to patient health literacy and can complicate patient understanding of their medical condition. The goal of communication is to allow for successful exchange of medical information to direct the physician in patient care. Clear and concise communication, providing thorough explanations, and summarizing the next steps are all strategies that help achieve this goal [2, 27, 29].

In our study, the least utilized communication strategies including the following: asking others’ point of view, encouraging questions, explaining actions of exam, introducing self, and shaking hands. Lack of using these communication skills did not seem to impact satisfaction with communication (CS score) or the caregiver perceived quality of communication (CPCQ score). The two communication strategies of introducing self and shaking hands are generally considered as part of building or establishing the patient-physician relationship [30, 31] with studies reporting patient expectations of these formal greeting strategies when meeting for the first time [32]. In our study, the lack of shaking hands or introductions was mostly observed in the follow-up clinic visits, but was also observed in 4 new patient visits. It is possible that the lack of formal greeting could be dependent on patient preference or other patient and provider factors. There is also literature reporting on the drawbacks from shaking hands including hygiene to limit the spread of pathogens [33], with one study suggesting that a fist bump may be a potential alternative [34]. Sill, others argue that avoiding shaking hands for the reason for limiting the spread of germs is not all that effective [35]. It is possible that with the rise in new modalities of communication and the presence of provider evaluations and patient testimonies available on the internet, that caregiver communication expectations are changing.

For the other 3 least utilized communication strategies, explaining actions of exam may not be viewed by the caregiver as important as there are also visual cues to explain what the physician is doing. Encouraging questions and asking other’s point of view are both important to help ensure that the patient is heard and that the physician can understand the patient’s perspective. However, it is possible that for patients who prefer a more authoritative clinical interaction, that encourage questions or asking about their point of view do not provide additional value to the communication experience. In our study, caregivers who preferred an authoritative approach but instead perceived a more collaborative communication experience during the clinical encounter, reported statistically significant lower quality of communication (CPCQ) and communication satisfaction (CS) scores. This observation suggests that understanding the patient or caregiver’s communication style and adjusting the communication delivery to meet this need is important. As such, including formal training on assessment of and strategies to meet the caregiver’s communication preference should be considered in the medical education setting.

Encouraging questions can be a strategy to help engage the patient in their care and to promote shared decision making. In our study, for the 16 encounters, in which the physician was observed not encouraging questions, the physician asked open ended questions in 13 of these encounters, which may have provided an opportunity for the caregiver to respond and communicate their concerns thus mitigating the need to solicit for the caregiver’s point of view or encourage additional questions. There was also no difference in the CPCQ score which suggests that despite the provider not utilizing some of these communication skills, that the caregiver still perceived receiving quality communication, which is the goal of the clinical encounter.

The communication strategies suggested by the different communication rubrics are intended as a guide but ultimately, the objective of a clinical encounter is for effective exchange of information between the patient or caregiver and physician. It is possible that indicators of effective communication may be changing as interpersonal communication modalities have adjusted.

Limitations of this study include the participants’ response bias in the surveys [36–38] as well as selection bias by physicians who were amenable to participation in the study. It is possible that despite responses being deidentified, that the potential of future interactions with the physician could have led to participants reporting higher CPCQ and CS scores than they actually perceived. In addition, having the clinic visit recorded could have disrupted a typical clinical setting and both physician and caregiver performance bias much be considered. In particular, being video recorded could have influenced physician communication behavior. Lastly, another potential limitation is the use of 3 research assistants to observe for communication strategies. However, research assistants were trained to have a standardized approach for assessing communication, but there could still be some variation in interpretation. Any discrepancy in assessment was resolved by discussion among the research assistants.

## Conclusion

In conclusion, this study demonstrates that physicians utilize many of the suggested communication strategies to help facilitate an effective clinical encounter in the pediatric ambulatory setting based on caregiver perception of the communication quality and satisfaction with communication. There were five universally used communication strategies across each encounter including: eye contact, good posture, speaking concisely, providing thorough explanations, and providing summary of next steps. The consistent utilization of these strategies despite different physician and caregiver communication styles suggests that these strategies may be integral to effective communication. Some suggested communication strategies were inconsistently utilized and it is possible that patient or caregiver communication needs may be adapting as there was no effect on communication satisfaction or quality scores. The concept of meeting caregiver communication expectations and the potential impact on the healthcare encounter can be further explored. Further studies are needed to evaluate effective communication strategies and to assess for changes in patient directed communication requirements, especially for caregivers in the pediatric setting.

## Data Availability

The authors confirm that the data supporting the findings of this study are available within the article.

## Abbreviations

ACGME: Accreditation Council for Graduate Medical Education
KEECC: Kalamazoo Essential Elements Communication Checklist
CPCQ: Caregiver Perception of Communication Quality
CS: Communication Satisfaction
SOLER: Sit squarely, open posture, lean towards the other, eye contact, relax
